# Residual β-cell function in Brazilian Type 1 diabetes after 3 years of diagnosis: prevalence and association with low presence of nephropathy

**DOI:** 10.1186/s13098-023-01014-z

**Published:** 2023-03-20

**Authors:** Monica A. L. Gabbay, Felipe Crispim, Sergio A. Dib

**Affiliations:** 1 Centre for Diabetes, Endocrinology Division, Department of Medicine, Escola Paulista de Medicina, Universidade Federal de São Paulo, São Paulo, Brazil; 2 Molecular Biology Laboratory, Endocrinology Division, Department of Medicine Escola Paulista de Medicina, Universidade Federal de São Paulo, São Paulo, Brazil

**Keywords:** C-peptide, Type 1 diabetes, Microvascular complications

## Abstract

**Background:**

Persistence of β cell-function in Type 1 diabetes (T1D) is associated with glycaemia stability and lower prevalence of microvascular complications. We aimed to assess the prevalence of residual C- peptide secretion in long-term Brazilian childhood onset T1D receiving usual diabetes care and its association to clinical, metabolic variables and microvascular complications.

**Methods:**

A cross-sectional observational study with 138 T1D adults with ≥ 3 years from the diagnosis by routine diabetes care. Clinical, metabolic variables and microvascular complications were compared between positive ultra-sensitive fasting serum C-peptide (FCP +) and negative (FCP-) participants.

**Results:**

T1D studied had ≥ 3 yrs. of diagnosis and 60% had FCP > 1.15 pmol/L. FCP + T1D were older at diagnosis (10 vs 8 y.o; p = 0.03) and had less duration of diabetes (11 vs 15 y.o; p = 0.002). There was no association between the FCP + and other clinical and metabolic variable but there was inversely association with microalbuminuria (28.6% vs 13.4%, p = 0.03), regardless of HbA_1c_. FCP > 47 pmol/L were associated with nephropathy protection but were not related to others microvascular complications.

**Conclusion:**

Residual insulin secretion is present in 60% of T1D with ≥ 3 years of diagnosis in routine diabetes care. FCP + was positively associated with age of diagnosis and negatively with duration of disease and microalbuminuria, regardless of HbA_1c_.

## Introduction

Type 1 diabetes (T1D) is an autoimmune disease characterized by progressive destruction of β cells that begins long before and continues long after the clinical diagnosis, as believed. It occurs at different rates among individuals, with some demonstrating long residual insulin secretion. There are controversies about the various factors related to the persistence of residual C-peptide secretion in T1D such as genetic, duration of diabetes and age at diagnosis among others. Ethnicity, for instance, might have influenced the rate of β cell loss after diagnosis since Hispanics had shown higher fasting C-peptide at the beginning of Trial Net New Onset Intervention Trials [1].

The importance of residual insulin secretion was already highlighted in the DCCT study (Diabetes Control and Complications Trial) in which participants with stimulated C-peptide higher than 200 nmol/L had better glycemic control, lower risk of hypoglycemia and lower risk of diabetes chronic complications [2, 3].

Classically chronological age, age at T1D diagnosis, duration of T1D, average systolic blood pressure and HbA_1c_ are associated with chronic diabetic complications, but the relation of latter with residual β-cell function has been shown heterogenous results [4–7], besides there is great evidence that C-peptide counteracts the detrimental changes causes by hyperglycemia at the cellular level in animal studies [8].

On the other side, there has no sufficient data about residual β cell function in long duration Brazilian T1D by routine diabetes care as in developing countries with a heterogeneous genetic population. These would help revealing the complex natural history of the disease considering its heterogeneity among different populations.

Therefore, the study aimed to assess the prevalence of residual C- peptide secretion, its association with clinical characteristics, and its impact on microvascular complication in Brazilian childhood onset T1D.

## Research design and methods

### Study participants

This is a cross-sectional observational study of 138 Brazilian people with T1D with more than 3 years of duration, selected from electronic medical records between 2014 to 2018 that had realized ultrasensitive C-peptide assay.

Inclusion criteria: Individuals recruited from the Diabetes Center—Federal University São Paulo (Southeast Brazil) with Type 1 diabetes (ADA criteria as continuous use of insulin since diagnosis, positive ketones, and presence of pancreatic autoantibodies).

Exclusion criteria: Hepatic disease, Chronic renal insufficiency or clinical characteristics suggesting another type of diabetes mellitus.

### Methods

The data analyzed were age (years), age at diagnosis of T1D (years), time of diagnosis of diabetes (years), sex, body mass index {BMI (Kg/m^2^)}, arterial hypertension (SBP > 130 mmHg e DBP > 90 mmHg, or > P95 for age), Total Cholesterol HDL-c (mg/dl), LDL-c (mg/dl), Triglycerides (TG)(mg/dl), TSH (mU/ml) and free T4(ng/ml), HbA_1c_ (HPLC—*high-performance liquid chromatography—*normal range 20–38 mmol/mol; 3.5 a 5.6%), and albumin excretion—Cobas Mira® Roche (AlbUCobas—NV < 17 mg/L or 30 mg/24hs).

The ultrasensitive C-peptide assay used was the Mercodia® ELISA (Uppsala, Sweden, cat. No. 10-1141-01) whose detection limit is 1.15 pmol/L, with an inter-and intra-assay coefficient of variation of 5.5% and 3.8%, for a C-peptide at 37 pmol / L. Participants whose FCP was greater than 1.15 pmol/L were classified as positive (FCP +) and below this value as negative (FCP-).

The presence of microvascular complications was taken derived of data from the electronic medical records and was fundoscopy for retinopathy, DCCT clinical exam protocol for neuropathy, microalbuminuria (in 2 of 3 albumin to creatinine ratios high > 30 mg/ml) for nephropathy and the evaluation of hypoglycemia was performed according to the frequency of file reported hypoglycemia.

### Statistical analyses

Normally distributed data were presented as mean SD and variable with skewed distribution were reported as median with interquartile ranges (25^th^,75^th^). Categorical variables are expressed as absolute frequency and percentage.

For the comparisons of two groups in continuous variables, the t-student test was used depending on the normality assumption (tested by the Anderson–Darling test) and for the others, the Mann–Whitney nonparametric test was used. Fisher’s exact tests were used for categorical variables. Simple and multiple logistic regression models were used to analyze the associations between the outcome variables. Statistics C was used to evaluate the model.

We applied a multivariable logistic regression model using complications event as the binary dependent variable and C-peptide as the independent variable. The duration-adjusted comparisons were represented by the P-value and odds ratio of the C-peptide term in the multivariable model. Scatter plots and bar plots were used to illustrate the distribution of variables. The level of significance was 0.05. Two-tailed hypotheses were considered. Software R version 3.6.0 was used to perform all analyzes.

## Results

One hundred and thirty-eight participants (58.7% men) were evaluated and 59.5% of them was FCP + (> 1.15 pmol/L). The means age (sd) at T1D diagnosis was 9.0 ± 0.47 years and by the time of the study was 22.0 ± 0.6 years. Diabetes duration of 12.0 ± 0.5 years and the mean HbA_1c_ was 8.35 ± 1.15% (67 ± 1.6 mmol/mol). Clinical, endocrine, and metabolic characteristics of the participants and the frequency of dyslipidemia and diabetic microvascular complications are shown in Table 1.

**Table 1:** Clinical, endocrine, and metabolic characteristics of T1D participants

Characteristics	Total	FCP−	FCP+	p value
N (%)	138	56(40.6)	82(59.4)	
Gender (male)%	58.7	57.1	59.8	0.8
Age at diagnosis (years)	9.0 ± 0.47	8.70 ± 5.14	10.28 ± 5.68	0.039
Age at recruitment (years) mean ± sd	22.0 ± 0.6	23.46 ± 7.22	21.84 ± 6.89	0.11
Diabetes duration (years) mean ± sd	12.0 ± 0.5	14.77 ± 6.71	11.41 ± 5.68	0.002
Hypothyroidism history (%)	16.0	17.9	14.6	0.2
BMI (Kg/m^2^) mean ± sd	23.00 ± 0.30	23.18 ± 3.26	23.52 ± 3.81	0.59
Insulin (IU/kg/day) mean ± sd	0.80 ± 0.10	0.80 ± 0.18	1.17 ± 2.11	0.08
HbA1c (%) mean ± sd	8.35 ± 1.15	8.55 ± 1.61	8.73 ± 1.58	0.42
LDL-C (mg/dl) mean ± sd	96.0 ± 25.0	97.4 ± 29.1	102.16 ± 29.55	0.6
HDL-C (mg/dl) mean ± sd	54.0 ± 13.0	56.64 ± 13.9	53.39 ± 13.0	0.1
TG (mg/dl) mean ± sd	73.0 ± 46.0	82.39 ± 41.8	90.00 ± 61.8	0.9
Dyslipidemia (%)	19.0	21.4	17.1	0.6
Hypoglycemia (%)	18.1	23.2	14.6	0.2
Nephropathy (%)	19.6	28.6	13.4	0.03
Neuropathy (%)	2.9	3.6	2.4	1.0
Retinopathy (%)	3.6	5.4	2.4	0.3

*FCP−* Negative Fast C-peptide, *FCP+ * Positive Fast C-peptide

*Negative C-peptide: < 1.15 pmol/L Positive C-peptide: > 1.15 pmol/L.

We observed that there was a statistically significant positive association between FCP + and age at T1D diagnosis (p = 0.039) and negative association (p = 0.002) with disease duration.

**Fig. 1:**
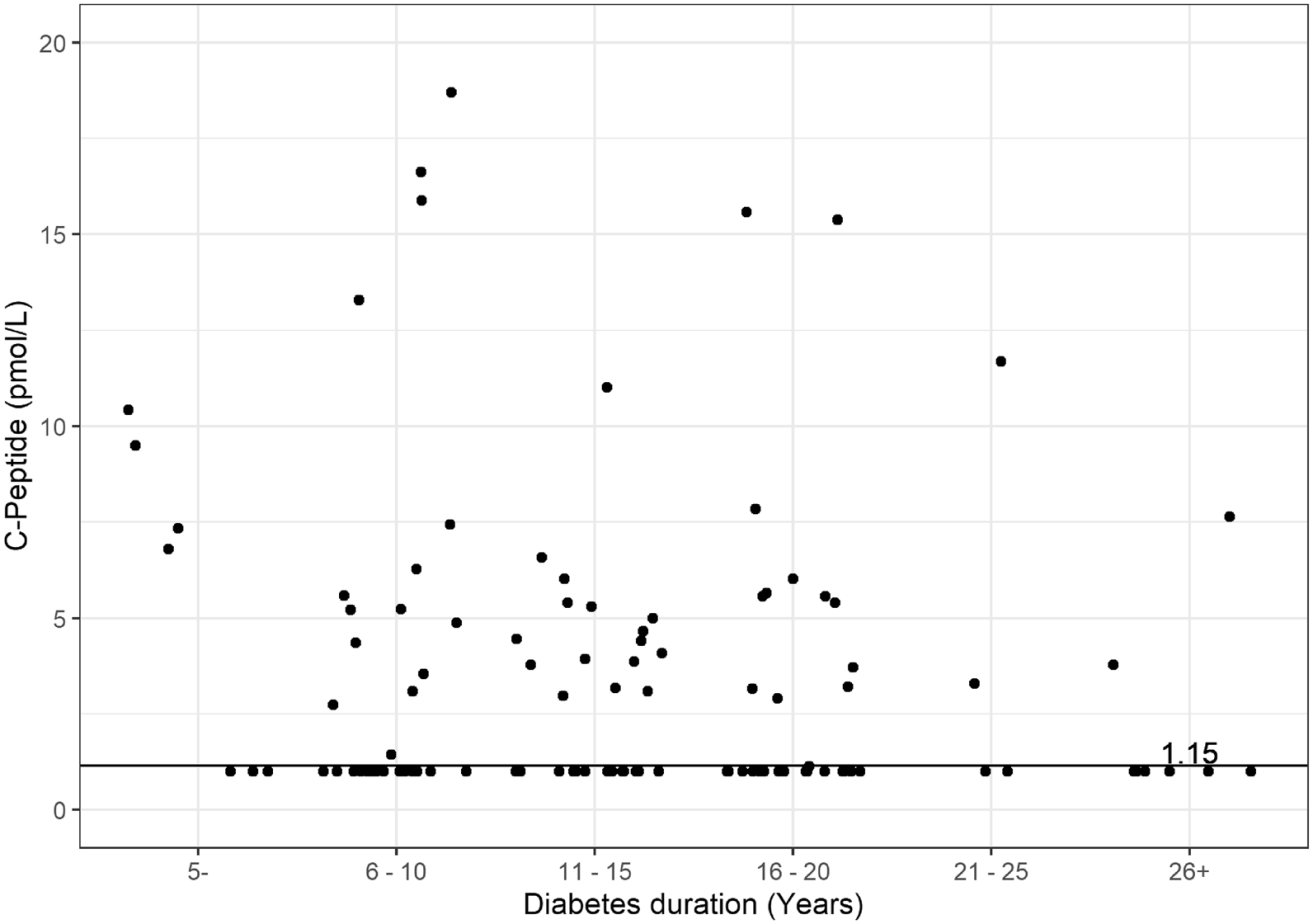
Fasting C-peptide levels according to diabetes duration in the T1D participants. The dashed horizontal line across the entire lower portion of the panel displays the limit of detection (1.15 pmol/L)

From the regression data, the probability most participants having FCP + with a diabetes duration of 5 years would be about 75%, in 10 years 64.5% and in 20 years less than 50% (Fig. 1). Also, these data shown that each time unit increase of diabetes duration(years) correspond to 8% reduction (OR = 0.92) in the probability of having FCP + .

There was no association between the presence of FCP and clinical (age and BMI) and metabolic variables (current HbA_1c_, LDL-c and TG) by the time of the study.

In a regression model adjusted for covariates, we found a lower prevalence (13.4%) of albuminuria among participants with FCP + compared to 27those with FCP- (28.6%, p = 0.031). Simple logistic regression showed that participants without nephropathy were 2.5 times more likely to have FCP + than those with this microangiopathy.

## Discussion

This study showed for the first time, that using an ultrasensitive method for serum C-peptide, around 60% of long-term Brazilian T1D with childhood-onset (~ 17 yrs of diabetes duration) had residual insulin secretion. These detectable FCP levels was associated with older age at T1D diagnosis and shorter diabetes duration. Importantly this group with even low levels of residual C-peptide had lower prevalence of microalbuminuria adjusted for diabetes duration and HbA_1c_ levels.

The decline of C-Peptide in T1D during the first years (in general three years) as reported in the literature is highly variable [9]. After this period patient become non-C-peptide secretor or low C-peptide secretor, which is more evident and prevalent when using ultra-sensitive C-peptide assays [10]**.** Most studies evaluating C-peptide in people with long-standing diabetes, included T1Ds with more than 3–5 years of duration, like one from Prof. Greenbaum group (an expert in this area) which use as an eligibility criteria of ages from 6 months to 46 years and diabetes duration of more than 3 years [5]**.** Others, such as the pioneering study by Denise Faustman [10], stratified by six intervals of disease duration included participants from 0 to more than 40 years duration. This is precisely why it has been studied in recent years, the persistence of prolonged C-peptide production and lower risk of complications, and that people diagnosed in adulthood have more insulin reserve than diagnosed in childhood. [11]. And a more recent study considered T1D individuals with diabetes duration with more than 5 years to evaluate C-peptide and complications [12]**.** Therefore, we selected participants with more than 3 years of diagnosis, but with an average diabetes duration time of 17 years, thus subject to the risk of complications. [13], mainly in our country where the T1D glycemic control legacy is not very good. However, the role of diabetes duration on fasting C-peptide in these patients was extensively studied and the probability of being C-peptide negative at 15 years of diagnosis is 45%, 20 years is 56%, 25 years 66%, 30 years is 75% and 40 years is 89%.

Preliminary studies, from UK Golden Years cohort [14], the 50-year Joslin Medalist study [15], and the Parisian cohort [16] have examined the clinical characteristics of type 1 diabetic patients with long disease duration. Subsequently, Joslin’s group further explored the data to evaluate possible markers of longevity without significant complications, including evaluation of C-peptide and some pancreatic histology. They showed that most of them could produce insulin endogenously (67% with standard C-peptide assay > 0.03 nmol/L) and confirm the presence of insulin in some available Medalist pancreases. [17] It is important to point out that many Medalists had monogenic diabetes variants that potentially contribute to heterogeneity of beta cell function in this group of patients.

In recent years, the development of an ultrasensitive assay for plasma C-peptide detection (change the lower limits of detection of approximately 50 pmol/L to levels as low as 1.15 pmol/L) has made it possible to detect residual secretion of β-cell function in people with T1D, even after decades of disease [18–20]. The percentage of residual FCP that we found is five times higher than the 13.2% found in a group of adults with T1D with a shorter disease time when using a regular C-peptide assay in routine clinics [21].

Studies with similar characteristics to our group, found 55–66% positivity (lower limit of 1.15 pmol/L for fasting serum C-peptide) and 52% for urinary C- peptide (> 30 pmol/L) [11, 15, 22] while others like participants of Exchange Clinic Network found detectable C-peptide in 29% among 900 participants, suggesting that residual C-peptide secretion is present in almost one out of three T1D individuals, three or more years from diabetes diagnosis [5]. (Table 2).

**Table 2:** Comparation of positive C-peptide among different studies using ultra-sensitive assay

Group (Country)	UNIFESP (BR)	Wang L, et al. [10] (USA)	Oram RA, et al. [29] (UK)	Davis AK et al. [5] (USA)	Rickels MR et al. [27] (USA)
N	138	182	74	919	63
Age (Mean or Median) (sd or range)	26 (6–52)	39(9–85)	16(9–23)	37.2 ± 18.9 (5–88)	18–65
Diabetes Duration (yr.) Median (range)	17 (3–34)	15 (0–73)	30 (19–41)	13 (3–81)	> 2
Assay sensitivity detection	Mercodia® 1.5 pmol/l	Mercodia® 1.5 pmol/l	Mercodia® 1.5 pmol/l	Tosoh Bioscience® 17 pmol/L	Tosoh Bioscience® 7 pmol/L
Positive C-peptide	FCP > 1.5 pmol/L 55%	FCP > 1.5 pmol/L 63%	FCP > 3.3 pmol/l 73%	Non-FCP > 17 pmol/L 29%	Peak in the MMT > 7 pmol/L 76%
A1c (%)	8.7 + 1.6	7.4 + 1.1	7.9 (7.2–9.0)	8.0 + 1.5	7.6 + 0.7

*FCP* fasting C peptide, *MMT* Mixed Meal Test

According to our analysis, we verified that one of the main factors related to the frequency of FCP considered positive (> 1.15 pmol/L) were age at diabetes diagnosis and the duration of the disease. These two factors, also shown in our population, are in according to other groups where age at the diagnosis was positively associated with C-peptide values [5, 10, 22]. The relationship between age at T1D diagnosis and residual C-peptide can be explained by different insulin profiles found at diagnosis as it has been shown in new onset teenage T1D that still retain approximately 40% of residual insulin-containing islets [21].

In our population each increase of one year of age correspond to 8% reduction (OR = 0.92) in probability of having FCP + . It would be expected better values ​​in our patients than the English and American ones which had longer disease duration, however our patients presented a marginally lower prevalence of residual C-peptide secretion [15, 17]. In addition, detectable C-peptide related to better A1c was found in 38% of children and adolescents after 10 years of diabetes compared to 24% of our patients as they are from public care services. Insufficient metabolic control may have contributed to this result. However, a recent systematic review and meta-analyses of all randomized controlled trials (RCTs) to preserve β-cell function in people with newly diagnosed T1D shown there is a lack of robust evidence that interventions to improve glucose control preserve β-cell function and efforts to treatment algorithms should be a priority [22].

However, one major debate today is what C-peptide level is clinically useful. In part because some studies that correlated residual C-peptide to chronic diabetes complications or hypoglycemia sometimes measure FCP [19, 23, 24] sometimes use stimulated C-peptide [21] besides the sensibility limit of ultra-sensitivity C-peptide assay used [23].

In our T1D participants with a mean 17 years of disease duration, using a C-peptide assay with a limit of sensibility of 1.15 pmol/L we did not find any association between residual C-peptide and lower HbA1c. This agrees with studies with similar T1D populations, while Trial net participants and T1D from the UNITED Team found this association only during the early course of diabetes [23, 25, 26]. Also, a recent work with young adult T1D with 5 years of diabetes duration demonstrated that only those with high levels of residual C-peptide (peak after stimulus test > 4.0 pmol/L or > 1.2 ng/mL) shown β cell responsiveness to hyperglycemia and likely contribute to glycemic control [25]. We have studied β cell responsiveness to sulfonylurea test in a sample of nine patients with FCP + and found that the maximum peak during the test was 0.286 pmol/L (data not shown) which is approximately 93% less than shown in the study above [27]. This might help understanding why we do not get an association between C-peptide levels and HbA_1c_ in our T1D group and why we did no find significant difference in the prevalence of hypoglycemia between our FCP + and FCP- groups. Nevertheless, heterogeneous relationship between the glucagon response to insulin-induced hypoglycemia does exist but again is most evident in T1D with high levels of residual C-peptide [25].

In relation to diabetes chronic complications and residual C-peptide in our study using FCP with limit of sensibility of the assay equal 1.15 pmol/L, we found an inverse relationship between the C-peptide reserve and albuminuria in FCP + T1D patients.

The frequency of albuminuria found in our participants was like people with T1D described in the literature with the same time of disease (19.6% vs 14.8%) where 65.2% of microalbuminuric patients had no detectable C-peptide. However, unlike predicted, we found no correlation of C-peptide and retinopathy, perhaps because of the low prevalence found in our participants (3.9% vs 9–40%) [28]. Also, maybe the low cut-off limit we used to define FCP + patients.

The classical work that found C-peptide protection from complications as nephropathy, neuropathy and retinopathy considered FCP levels > 10 pmol/L (with the same C-peptide assay that we used) almost 9 times higher than our C-peptide cut-off. Other study [20] using the same cut-off value of ours (1.15 pmol/L) also did not find relation among residual C-peptide and retinopathy, neuropathy but marginally lower macroalbuminuria in those with detectable levels (23.4% vs 0%, p = 0.07). A larger sample size might have allowed a bigger difference [25].

One point for discussion is why these low levels of C-peptide can decrease the prevalence of nephropathy, regardless to HbA_1c_ level but not the two others microangiopathies (retinopathy and neuropathy).

Diabetic nephropathy (DN) is one of the major microvascular complications, present in 20 to 40% of T1D people and is one of the most important causes of kidney failure (KF), but the rate of renal decline varies widely among them. On the other hand, in addition to its well-known role as a biomarker of functional beta-cell mass, the C-peptide is a bioactive molecule with physiological effects on peripheral cell targets and with antioxidant protection on vascular endothelial. Small trials in which C-peptide was given to subjects with T1D with nephropathy or neuropathy showed that C-peptide mitigates renal and neuronal complications [30, 31], while others had shown that C-peptide can suppress various molecular mechanisms involved in the pathophysiology of DN and therefore could prevent the onset and progression of KF [32]. Interestingly almost two decades ago, a double-blind randomized study had demonstrated that administration of biosynthetic human C-peptide plus insulin reduced glomerular permeability and urinary excretion of albumin [33].

In relation to the lower prevalence of DN in our FCP + T1D regardless of the same HbA1c as FCP- ones, we can speculate that the former could have less glycemic variability**.** Since recent works have been shown that for every 100 pmol/L increases in C-peptide peak the percentage of time spent in the range (70–180 mg/dl) increased by 2.4% with a reduction in time spent at level 1 hyperglycemia (> 180 mg-dl) and level 2 hyperglycemia (250 mg-dl) by 2.6% and 1.3% respectively [34]. Is important to mention that lower time in range was associated with presence of composite microvascular complications in recent study with a group of adults with long T1D duration [34].

When we studied the relationship between C-peptide levels and nephropathy in our T1D group we found out that all patients with fasting C-peptide ≥ 46.9 pmol/L (0.14 ng/ml) were negative for this microangiopathic complication. This number is close to cut-off used in most studies (50 pmol/L but one that considered 10 pmol/) [33] for identify patients at higher risk for complications, more frequent and great glycemic excursions and low 1,5 AG levels.

Finally, at this level of glycemic control, we did not find difference on lipid profile between T1D patients with and without residual C-peptide. However, results in this area are heterogenous, some works showing better lipid profile in C-peptide positive and others showing no relationship between unstimulated C-peptide values and lipid parameters in either remitters or non-remitters T1D adult [35].

The present study has some limitations as the small sample size, the cross-sectional data, using FCP although the literature has already shown a good correlation with stimulated C-peptide. Another limitation was the low prevalence of chronic diabetes complications that may have impaired the association studies. The strengths were the assay sensitivity, the heterogenous genetic background of our T1D population and the real-world data from their routine treatment.

We can conclude that most of our T1D participants, like American and European data, have residual beta-cell function demonstrated with the use of an ultrasensitive assay after a decade of disease, and this minimal detectable C-peptide appears to protect against albuminuria regardless of HbA_1c_.

Finally, the importance of persistent beta-cell residual function reinforces strategies for its preservation since diagnosis and suggests that a significant percentage of patients with T1D, even after decades of diagnosis, may have benefits in slowing the development of diabetes nephropathy.

## Data Availability

All data are available.

## Abbreviations

T1D: Type 1 Diabetes
HbA_1c_: Glycated Hemoglobin A1c
BMI: Body Mass Index
SBP: Systolic blood pressure
DBP: Diastolic blood pressure
TG: Triglycerides
